# Utilizing the Style Under Stress™ tool within Crucial Conversations© methodology to stimulate veterinary students' reflective practice of communication behaviors during client interactions

**DOI:** 10.3389/fvets.2026.1746568

**Published:** 2026-02-04

**Authors:** Rodney S. Bagley, Amelia Mindthoff

**Affiliations:** 1 Department of Veterinary Clinical Sciences, College of Veterinary Medicine at Iowa State University, Ames, IA, United States; 2 Office of Academic and Student Affairs, College of Veterinary Medicine at Iowa State University, Ames, IA, United States

**Keywords:** crucial conversations, silence, student perspectives, style under stress, violence

## Abstract

**Introduction:**

“Crucial” conversations occur commonly in the veterinary profession and students require exposure to, and training in, these difficult communication dynamics.

**Methods:**

To introduce the concept of how people respond during perceived crucial, intense, or difficult communication dynamics, we asked first-semester veterinary students to complete a “Style Under Stress™” self-assessment offered by the Crucial Conversations© (Crucial Learning) educational platform. This tool is used in the training of the Crucial Conversations© model and provides important insight into how people may respond during crucial conversations. First-year veterinary students in the initial (fall) semester of their curriculum completed this assessment beginning in 2016 (Class of 2020) continuing annually through fall of 2024 (Class of 2028).

**Results:**

Responses were compared between classes (years) to determine student style consistency between years. Most students were assigned a “Silence” style (examples including “withdrawing,” “masking,” or “avoiding”) and this was preserved throughout the different years.

**Discussion:**

The feedback provided through the assessment is used as a component of medical communication training in developing competency during crucial communication encounters. Students learn about their individual defaults as well as how others may respond in similar crucial conversation situations.

## Introduction

Medical communication can be challenging under certain circumstances and having “crucial” conversations is very common in the veterinary medical profession. A crucial conversation implies an interpersonal interaction that is important, necessary, and significant. Often there are additional connotations with these interactions being described as “difficult,” “hard,” “emotional,” “uncomfortable,” or “intense” and commonly revolve around delivering “bad news” to owners or agents (1–3)^1^. Within the communication spectrum these types of interpersonal communication dynamics are some of the most challenging for students to experience, practice, and master. A foundational aspect of learning to navigate these interactions is determining how one is likely to respond to an emotionally charged or challenging conversation. Based on individual constructivist learning theories and adult learning theories, utilizing existential experience and self-assessment in learning activities can support exploring personal psychological defaults that influence situational outcomes (4–12). This self-knowledge helps students reflect on themselves through their self-assessment of how they react to difficult, uncomfortable, and/or crucial conversations. Building on this knowledge, students can next practice developing skills to counterbalance these tendencies and ultimately transpose this self-knowledge to predict how others may react under similar circumstances.

A self-reflective learning paradigm exploring how one reacts to difficult, intense, challenging or uncomfortable communication dynamics is found within the Crucial Conversations© (CC) learning platform (2, 3)^1^. The Crucial Conversations model upon which the learning platform was first described in book form in 2012 and was expanded through the years into a comprehensive learning strategy for having crucial conversations. This model is currently supported within the Crucial Learning platform^1^. This platform provides foundational principles related to establishing psychological safety in communication dynamics. The original research underpinning this model was collected from United States multinationals and in a United States work context^1^. Therefore, the applicability of this material is relevant in English-speaking environments but may require further adaptation and assessment in other communication environments. Context should be acknowledged when applying these concepts globally and with an appreciation of cultural background and the lived experience of those involved in the interaction (13). As the communication principles in this model focus on self-awareness, self-regulation, and self-control, it seems reasonable to explore these training activities in challenging human interactions in a veterinary medical environment.

There are various foundational tenets described in CC methodology (2, 3)^1^. In this platform, a “crucial” conversation involves three elements: “high-stakes,” ‘high emotions,” and most importantly, “opposing perspectives.” These three elements are actualized in a veterinary medical context in numerous human-to-human interactions. Veterinary medical situations are often “high-stakes” and can involve life/death decision-making. Many interactions involve “strong emotions” such as fear, sadness, and anger. Additionally, emotions are known to influence medical decision-making (14). The “opposing perspectives” is the fundamental conflict element. This may be as simple as the veterinarian possessing medical knowledge that the owners do not have with an inherent knowledge power difference between the veterinarian and the client or as complex as true disagreements related to medical care and management. Situations where clients disagree with the veterinarian on issues ranging from the cost of medical care to the best treatment options obviously fulfill the criteria of a “crucial” conversation dynamic. Therefore, the CC principles of framing, understanding, and participating in these crucial conversations seem a reasonable method to utilize for communication training of veterinary medical professionals.

A foundational concept of the CC model is that to have effective dialogue (that is, a free exchange of information within an interaction), the individual participating in that interaction needs to feel “safe” (2, 3)^1^. “Safe” in this context is feeling that the individual can share their thoughts, ideas, opinions, and perspectives freely and openly in the conversation without fear of negative consequences (15–22). Each individual ultimately determines whether they feel safe in a conversation and for a variety of reasons. Often this learned assessment is based on previous experiences where safety has been violated by another. Based on the CC model, when individuals do not feel “safe” for any reason in having a free and open exchange of information or dialogue they tend to default to two general protective dichotomous strategies in response: a “silence” style or a “violence” style (Table 1) (2, 3).

**Table 1:** Definitions and example of “Style under stress” behaviors.

**Silence**	**Any act to purposely withhold or not share information**		
	**Subcategories**	**Description**	**Examples**
	Masking	Selectively communicating inaccurate opinions	Sarcasm, “sugarcoating,” “couching”
	Avoiding	Attempting to deflect	Changing the subject
	Withdrawing	Physically silent or leaving the environment	Physically not saying anything
**Violence**	**Any act to compel others to your point of view**		
	Controlling	Coercing or forcing your views on others	Dominating the conversation, interrupting, overstating information, speaking in absolutes, changing subjects, or using directive questions, using hyperbole
	Labeling	Placing a verbal label on others or ideas to dismiss them under a general stereotype or category	Naming in a derogatory manner
	Attacking	The motive is to make others suffer	Belittling, threatening

A silence style is any act to purposely withhold or not share information (2, 3). Most commonly this is manifested as remaining silent during a conversation. The outcome of this strategy is to remain silent even to the point of physically leaving the communication dynamic. The basic motivation of this strategy is to not share information with others participating in the dialogue through lack of verbal channels. The behaviors that are characteristic of the “silence” style include actions such as “withdrawing,” “masking,” or “avoiding.” These silence strategies are actualized by selectively communicating accurate information (masking), attempting to deflect (avoiding), and physically remaining silent or leaving the dialogue (withdrawing). The intent of these behaviors is not to actively provide false or inaccurate information, but rather to hide one's “true feelings.”

Importantly, the “violence” style in this model is not a physically violent one, but rather a “violence” communication style (2, 3). The behaviors that are characteristic of the “violence” communication style include “controlling,” “labeling,” and “attacking.” “Controlling” may include competitive interrupting, increasing voice volume, dominating the conversation time through over-speaking, overstating, speaking in absolutes, or changing subjects without the consent of the others involved in the dialogue. “Labeling” is applying a negative descriptor (i.e., a “label”) to the other such as “wrong,” “dumb,” “stupid” or even creating a new descriptor for that individual. Using this label or descriptor is interpreted by the individual being named as a “negative” descriptor and therefore, derogatory. Another strategy in the “violence” style is using hyperbole by using absolute words such as “everybody” or “nobody” to describe levels of agreement. Finally, “attacking” includes actions such as “belittling” and “threatening.”

Within the CC learning activities there is an assessment instrument determining one's “Style under Stress™” which helps individuals self-assess their likely default communication strategy when feeling “unsafe” in a dialogue (23). This assessment is not intended as a traditional validated personality or similar instrument and should be viewed more in line with a training tool. The “Style under Stress” exercise, however, does allow for self-reflection on reactions to uncomfortable communication dynamics and, as importantly, provides a lexicon to frame associated cognitive states that are sometimes difficult to verbalize. Other advantages of utilizing this tool for learning are there is no financial cost to complete, easily accessible online, time-efficient, and provides feedback on the individual's default style and how that style might manifest during a crucial conversation.

In introducing veterinary students to aspects of skill development for difficult communication dynamics, we ask first-year veterinary students to complete the “Style Under Stress” assessment from the Crucial Learning platform (also previously known as Vital Smarts) (23). Our initial interest in utilizing this exercise was to empirically determine what students' default strategies would be during a “crucial conversation” in preparation for future crucial conversations. With knowledge gained we then discuss the concepts of establishing communication “safety” in a follow-up lecture in this same course. We amplify these concepts with additional explicit communication dialogue and learning opportunities in the third-year preclinical core course dedicated to medical communication in order to longitudinally engage students in thinking about, contemplating, and explicitly discussing the various aspects of having crucial conversations as they prepare to enter the veterinary profession. The CC model additionally provides a common lexicon to help individuals understand components of the communication dynamics associated with crucial or difficult conversations (24). The learning objectives during the post-completion discussion of this assignment are to:

1) Explain differences in communication styles and preferences when individuals feel unsafe during a communication interaction.
2) Develop self-awareness of one's own communication preferences under similar circumstances.
3) Utilize this knowledge when communicating with others to explicitly provide a “safe” communication environment.

Given that we have been using this tool for several years for self-reflection and to prompt dialogue related to communication dynamics, we retrospectively analyzed the data we have collected to determine if these default communication strategies have changed or remained consistent (preserved) over time.

## Methodology

The course assignment is provided in a core introductory course to the veterinary career colloquially entitled “Careers and Career Success” in which students are introduced to various aspects of the veterinary career and are equally provided self-reflective assignments related to various aspects of the veterinary profession. This assignment is a component of developing communication as a competency articulated within the AVMA Council on Education's core competencies.

For this assignment, students are provided with two hyperlinks with activities to complete through our learning management model. The first is the link to the assessment through the Crucial Learning platform (https://cruciallearning.com/style-under-stress-assessment/) (23). In this assessment the students are asked to identify both a relationship and a circumstance where there is/has been a disagreement between themselves and another. With this context, the instrument has 30 questions with true/false forced-choice answers to be selected. The assessment is usually completed in less than 15 min, however time to completion is dependent upon how much contemplation time the student takes on each question. Following submission of the answers there is formal feedback provided from Crucial Learning on how often one defaults to either a “silence” or “violence” strategy. We do not ask the students to share the actual individual feedback they received from the assessment; however, we ask that they provide us anonymously with their default style and associated subcategories. The second is a link to a Qualtrics survey (https://www.qualtrics.com/) with two associated questions to answer from the feedback they receive after completing the assessment:

1) My “Style under Stress” is: (“silence” or “violence”) (forced choice based on their results)
2) Of the following characteristics of my “Style under Stress” I most commonly do what (choose all that apply)?

Masking, Avoiding, Withdrawing, Controlling, Labeling, Attacking

We use results to assess the general class responses and to compare each class results relative to previous classes to determine trends over time. For this study the collective student responses in this educational activity were submitted to Iowa State University Human Subjects Institutional Review Board review (IRB 23-381, amended November 5, 2024, to include fall 2024 student data) and deemed exempt.

### Analysis

For each Cohort, student responses were collected during the fall of their first year in the curriculum during November which was approximately, depending on the year, the 9th to 11th week of their veterinary curriculum. As such, the year presented in analyses refers to the respective cohort's initial year in the veterinary curriculum.

The percentage of students who recorded either the silence or violence style (by academic year) was collated by dividing the total in each category by the total number of students who completed the assignment for that academic year. To determine whether the odds that collective individual styles changed over time, we employed logistic regression with academic year set as a categorical predictor.

### Subcategories

We examined the frequency with which students indicated that they were assigned to one or more of the following six subcategories: controlling, masking, avoiding, withdrawing, labeling, and attacking. These categories were not mutually exclusive as students could be assigned to more than one category.

To determine the effect of academic year on subcategory presence, we conducted two sets of *post-hoc* analyses: (1) comparison of each academic year to the initial year of data collection (i.e., 2016); and (2) comparison of each academic year to the preceding year (e.g., comparison of 2017 to 2016, of 2018 to 2017, etc.). To determine whether the frequency by which the subcategories that emerged varied by academic year, we conducted a logistic regression analysis for each of the subcategories (categorical predictor: academic year; outcome: presence/absence of the subcategory).

## Results

The number of students who completed the assignment varied per year based on class size (between 117 and 151 per year). In total, there were 2,015 individual students who provided their responses for this analysis. As this was a core class assignment, there was no dropout rate as all students in each class completed the assignment.

### Student general demographics

We did not match individual student demographics or other individual identifiable information with individual responses. Students' names were subsequently removed from the data set before analysis and we did not correlate any demographic data and the responses. For context relative to the cohort demographics, however, we provide the age and gender distributions for each of the classes within our sample as self-reported from admissions data collected at the time of matriculation into the first semester of the veterinary curriculum (Table 2). Gender distribution did not greatly vary across the years, with most students in each of the classes identifying as female (81.7%−85.8%, depending on the class year). The average age of students was consistent across the classes as well (23.5–24.4 years of age). As we did not identify individual answers by gender and age across the classes, we did not include these variables as covariates within our analysis. In addition to this assignment, in a separate assignment, students were asked about their planned practice species (Table 3). Again, this information was not directly linked to the results data in this study, and we made no correlation of species of interest and the style under stress assessment outcome. This information is presented here for additional context of the general features of the Cohort.

**Table 2:** Demographic information from each cohort.

**Year**	**Gender**		**Age**	
	**Male**	**Female**	**Average**	**Range**
2016	18.1%	81.9%	23.7	21–42
2017	14.8%	85.2%	24.0	21–47
2018	14.1%	85.2%	23.8	20–53
2019	16.8%	83.2%	23.9	20–40
2020	17.7%	82.3%	24.4	20–46
2021	18.3%	81.7%	23.5	20–35
2022	17.3%	82.7%	24.2	20–44
2023	14.2%	85.8%	24.3	20–49
2024	17.3%	82.7%	24.1	21–42

Self-identified data. For both 2018 and 2024, one student declined reporting their gender.

**Table 3:** Percentages of students' anticipated career choices.

**Anticipated career choice**	**2016**	**2017**	**2018**	**2019**	**2020**	**2021**	**2022**	**2023**	**2024**
Companion or companion mixed	NA	30%	31%	32%	37%	40%	44%	37%	41%
Equine	NA	3%	5%	7%	7%	4%	6%	4%	10%
Food animal or food animal mixed	NA	24%	34%	21%	21%	20%	18%	21%	20%
Mixed	NA	34%	29%	32%	26%	30%	26%	30%	26%
Exotics/other	NA	9%	1%	8%	10%	6%	6%	9%	1%

### Change by year in “Style under Stress” outcomes

**Figure 1:**
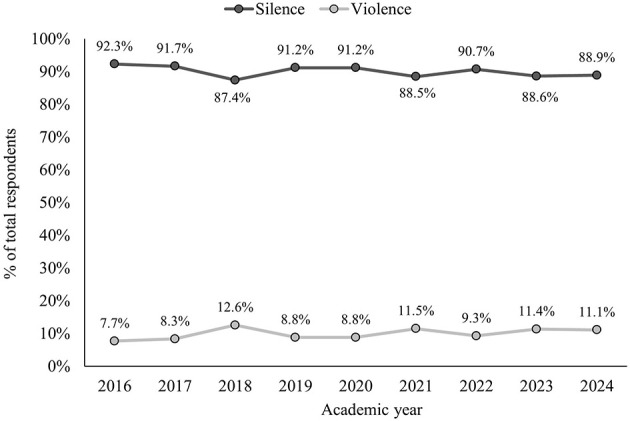
Percentage of students who were assigned “silence” or “violence” for the “Style Under Stress” assessment, by academic year.

Displayed in Figure 1 is the percentage of students who were assigned to either a “silence” or “violence” category, by academic year. Descriptively, most students defaulted to the “silence” category. The academic year did not significantly predict the silence or violence category that students received, χ^2^(7) = 3.2, *p* = 0.860.

**Figure 2:**
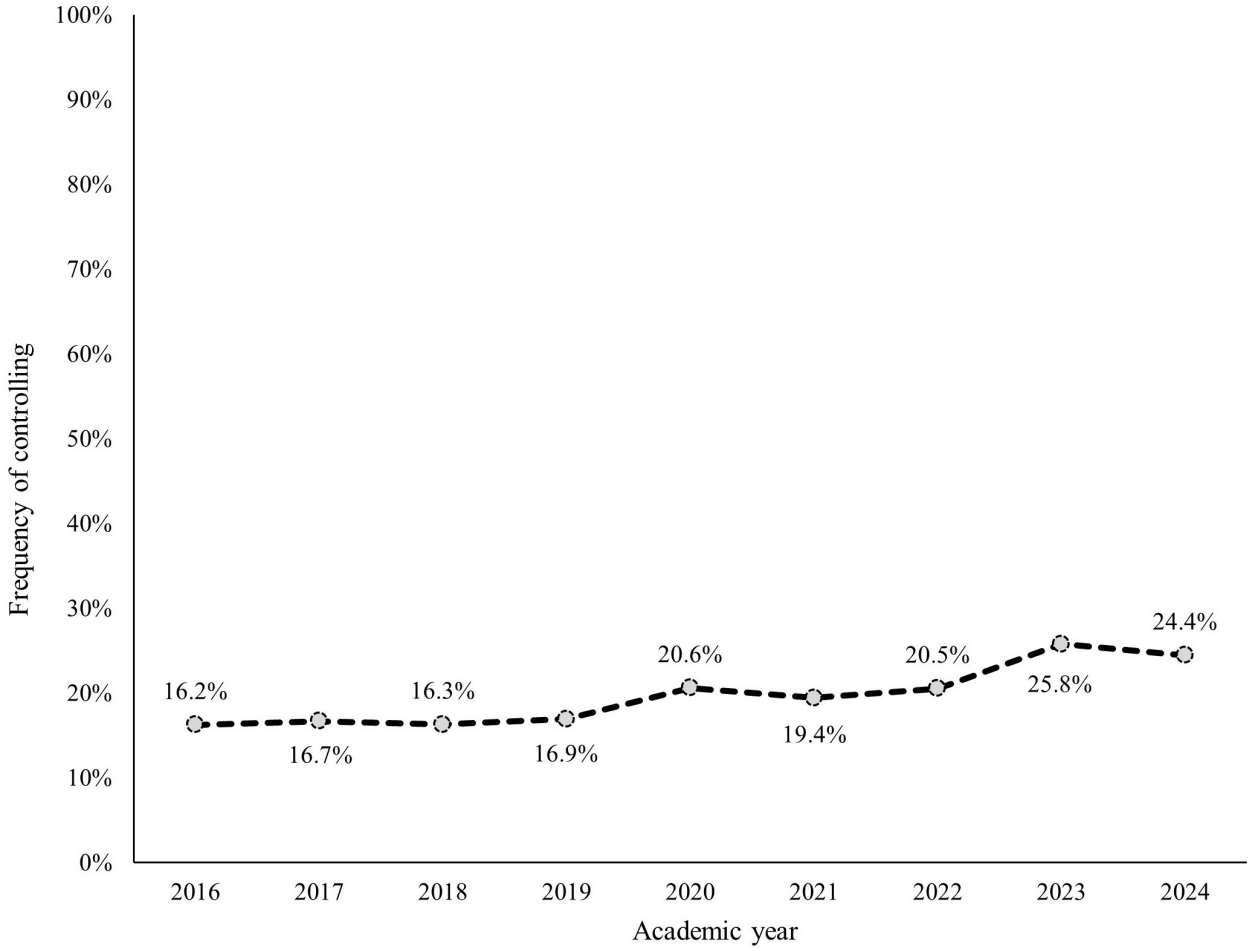
Percentage of students who were assigned the controlling descriptor, by academic year.

**Figure 3:**
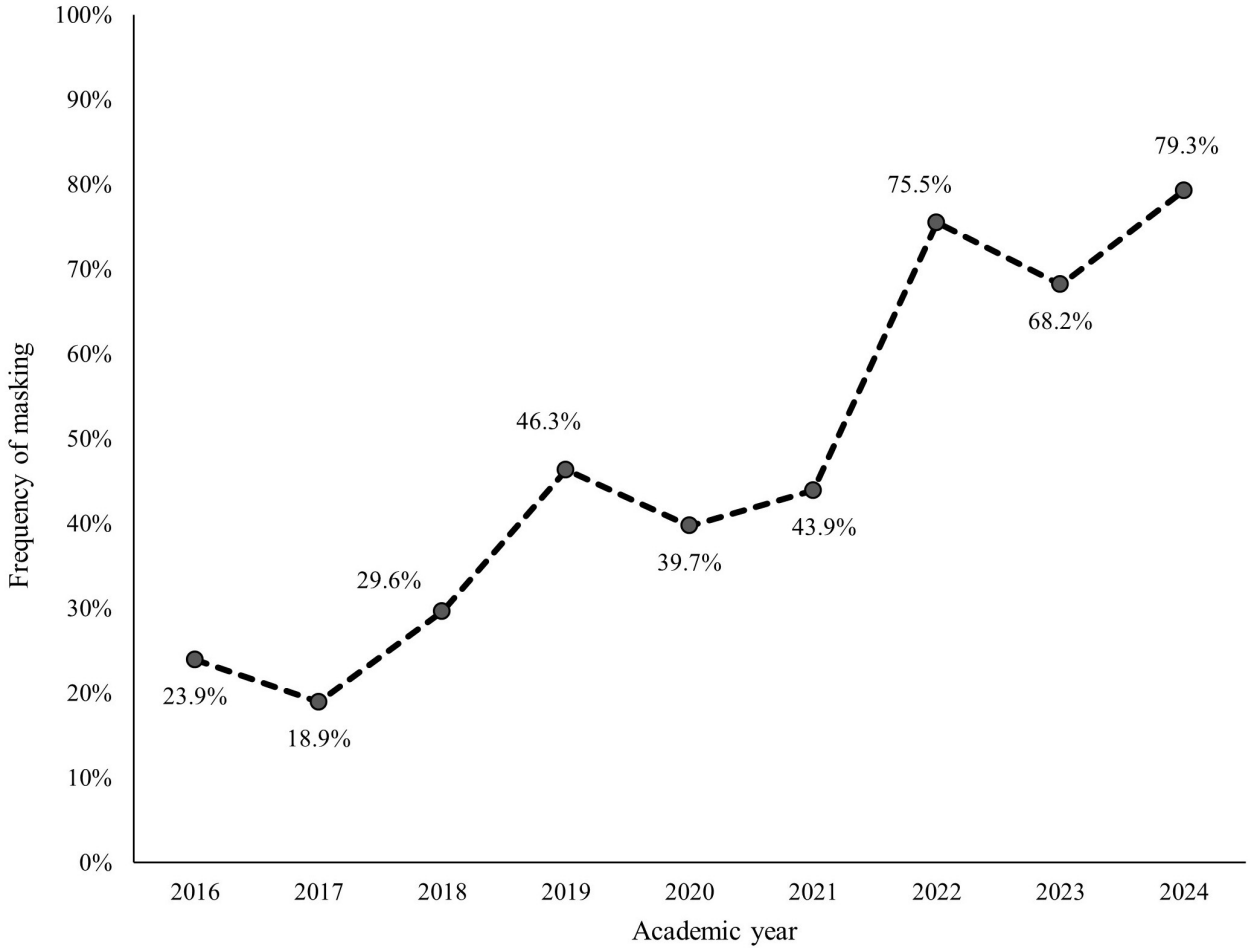
Percentage of students who were assigned the masking descriptor, by academic year.

**Figure 4:**
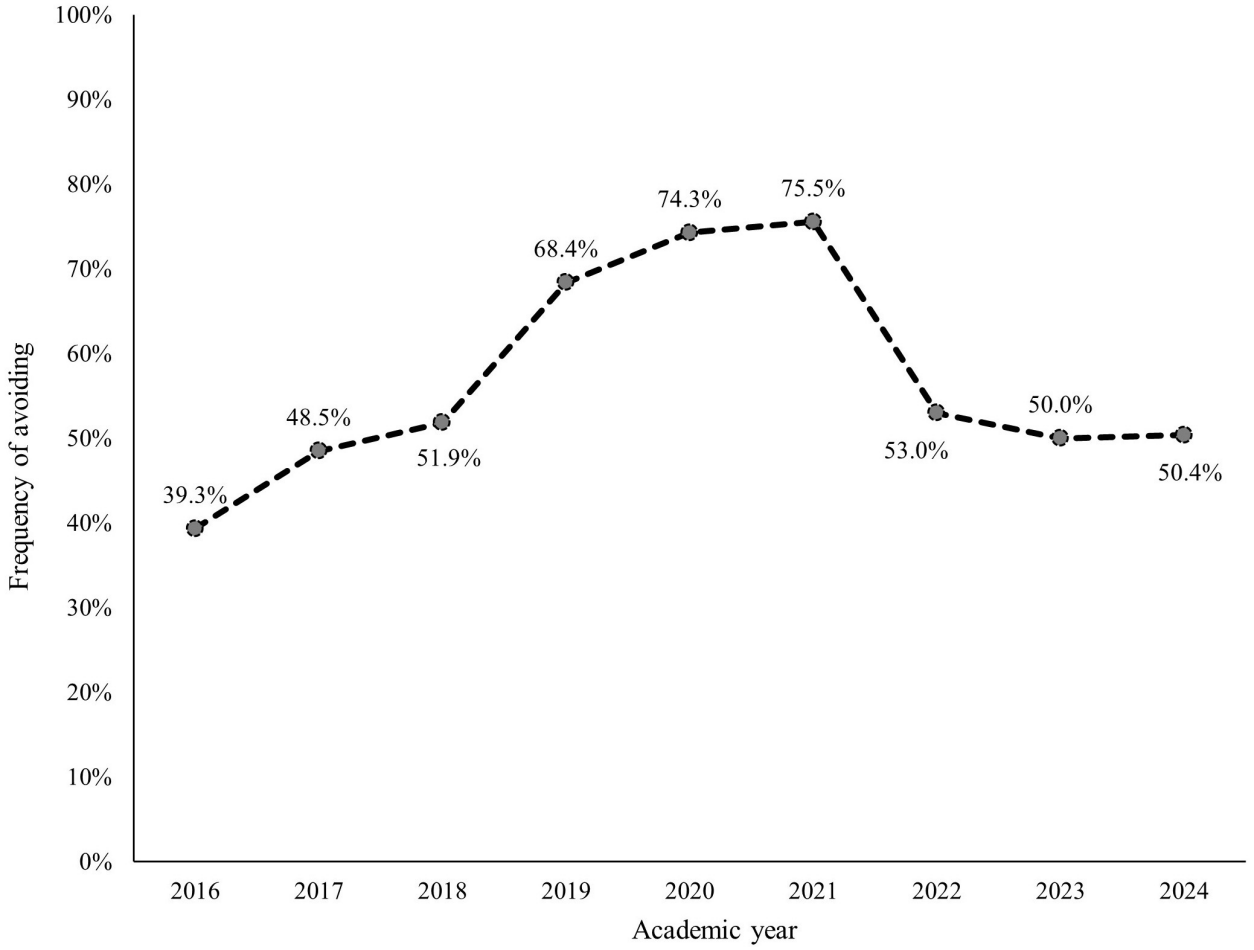
Percentage of students who were assigned the avoiding descriptor, by academic year.

**Figure 5:**
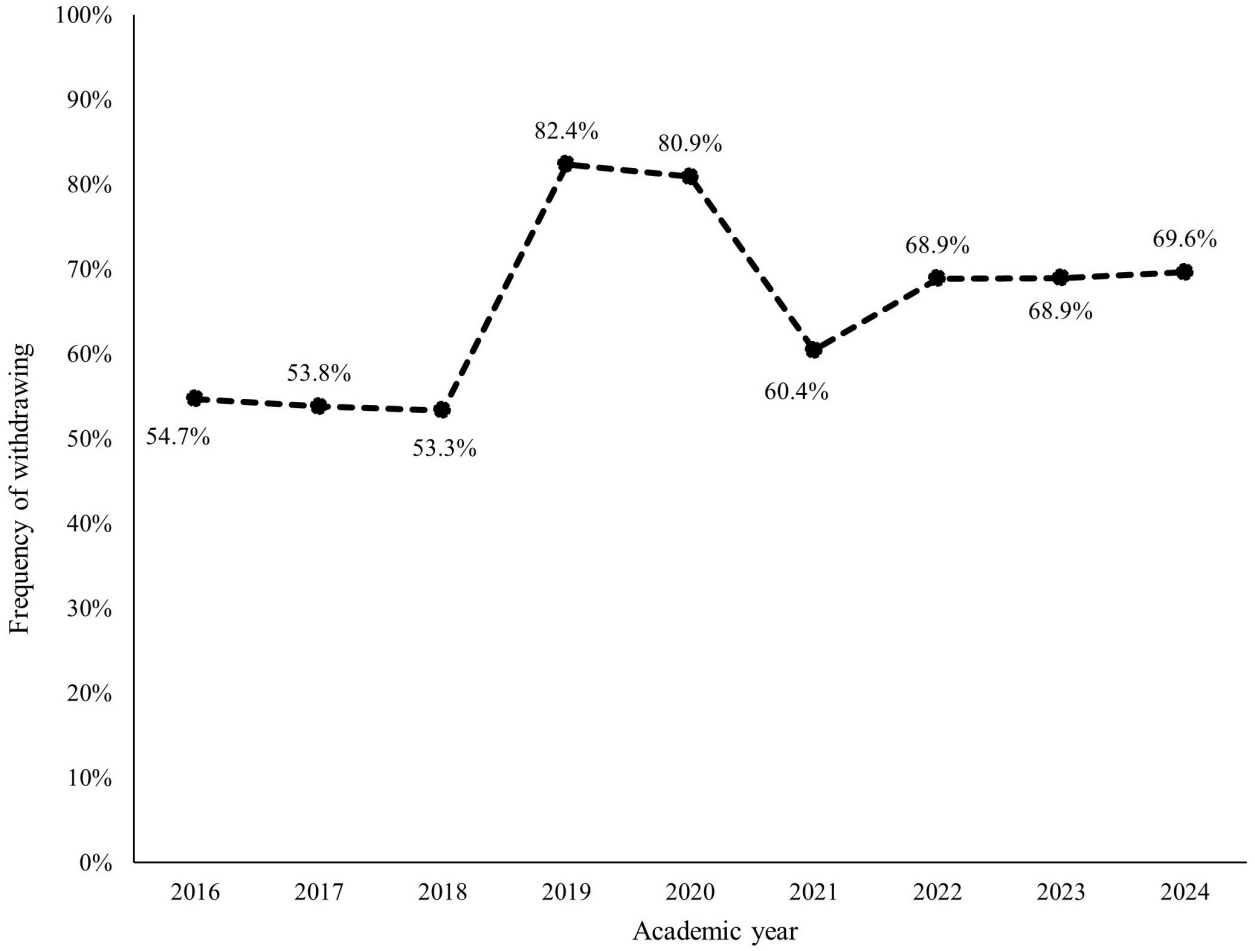
Percentage of students who were assigned the withdrawing descriptor, by academic year.

**Figure 6:**
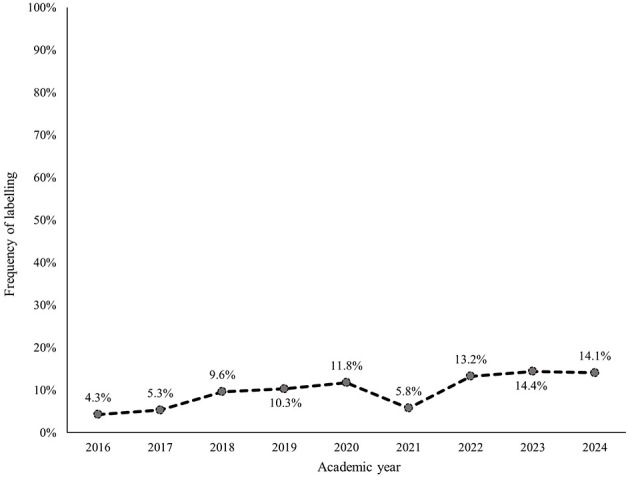
Percentage of students who were assigned the labeling descriptor, by academic year.

**Figure 7:**
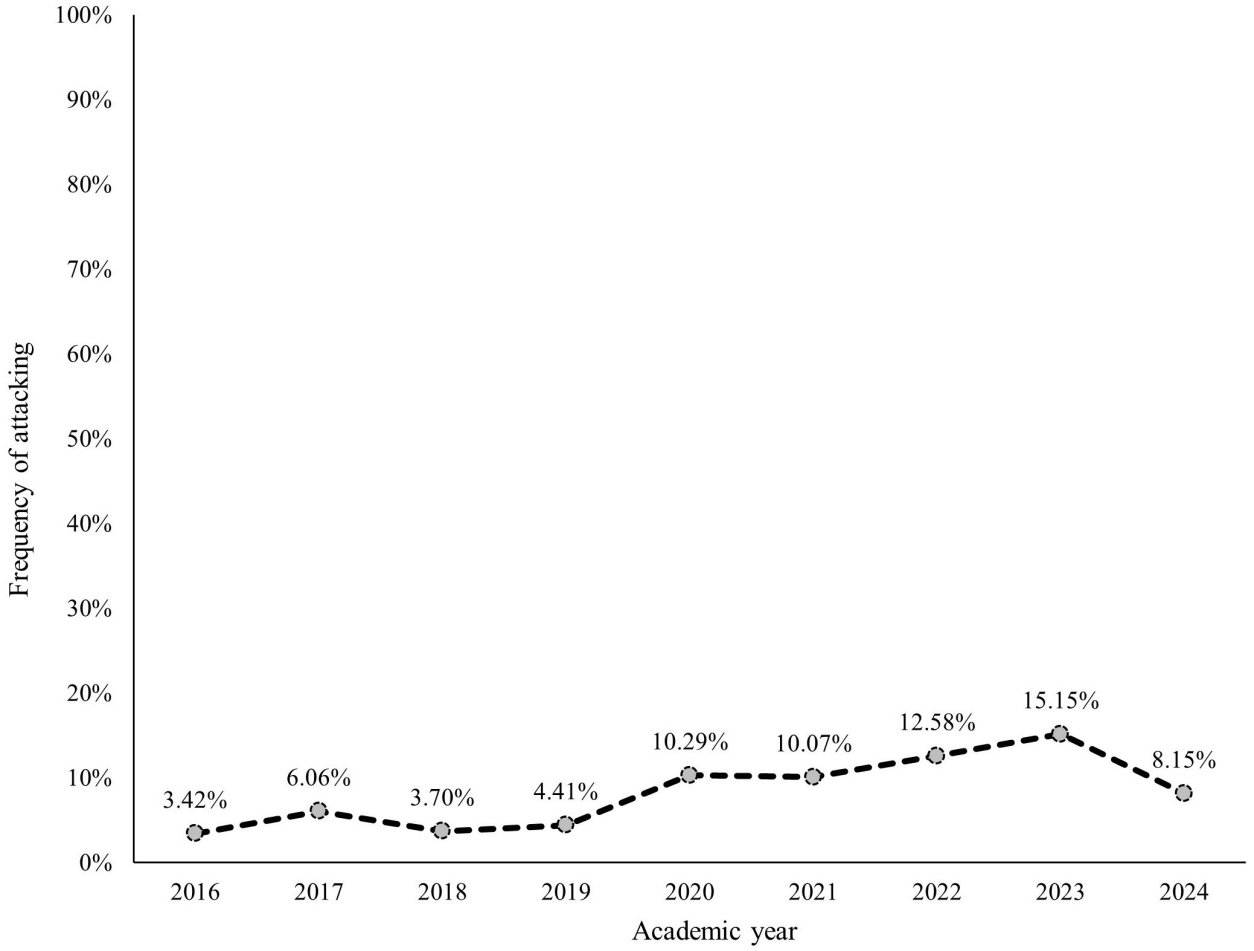
Percentage of students who were assigned the attacking descriptor, by academic year.

Within each subcategory, avoiding, withdrawing, and masking were most frequently identified (Figures 2–7). The odds of students being characterized by masking, withdrawing, and labeling were significantly higher in more recent years compared to the initial year of data collection (Table 4). Additionally, the masking, withdrawing, and labeling subcategories demonstrated significant increases in odds from certain academic years to the next (Table 4). The academic year significantly predicted the presence of all subcategories, except for the controlling subcategory.

**Table 4:** Results for logistic regression analyses examining whether academic year predicts the presence/absence of the six subcategories.

**Result**	**Controlling**	**Masking**	**Avoiding**	**Withdrawing**	**Labeling**	**Attacking**
**Wald test**	*χ^2^***(7)** = **6.3**, ***p*** = **0.500**	*χ^2^***(7)** = **140.9**, ***p***<**0.001**	*χ^2^***(7)** = **64.4**, ***p***<**0.001**	*χ^2^***(7)** = **54.6**, ***p***<**0.001**	*χ^2^***(7)** = **14.8**, ***p*** = **0.038**	*χ^2^***(7)** = **21.7**, ***p*** = **0.003**
***Post-hoc*** **: Reference group set to 2016 (initial year of data collection)**						
**2016 vs**.						
2017	1.03 [0.53, 2.03]	0.74 [0.40, 1.36]	1.45 [0.88, 2.41]	0.96 [0.58, 1.59]	1.25 [0.39, 4.34]	1.82 [0.56, 6.98]
2018	1.00 [0.51, 1.98]	1.34 [0.76, 2.37]	1.66 [1.01, 2.76]^a^	0.95 [0.58, 1.56]	2.39 [0.87, 7.63]	1.09 [0.28, 4.48]
2019	1.05 [0.54, 2.06]	2.74 [1.61, 4.77]^c^	3.34 [2.00, 5.64]^c^	3.87 [2.20, 6.94]^c^	2.57 [0.95, 8.16]	1.30 [0.36, 5.21]
2020	1.34 [0.71, 2.58]	2.09 [1.22, 3.65]^b^	4.45 [2.63, 7.67]^c^	3.50 [2.02, 6.21]^c^	2.99 [1.13, 9.37]^a^	3.24 [1.12, 11.70]^a^
2021	1.24 [0.65, 2.40]	2.49 [1.46, 4.31]^c^	4.77 [2.81, 8.23]^c^	1.26 [0.77, 2.08]	1.37 [0.44, 4.64]	3.16 [1.10, 11.41]^a^
2022	1.33 [0.71, 2.54]	9.79 [5.65, 17.47]^c^	1.74 [1.07, 2.85]^a^	1.83 [1.11, 3.03]^a^	3.42 [1.34, 10.54]^a^	4.07 [1.48, 14.34]^a^
2023	1.79 [0.96, 3.40]	6.81 [3.93, 12.10]^c^	1.54 [0.93, 2.56]	1.84 [1.10, 3.10]^a^	3.77 [1.46, 11.67]^a^	5.04 [1.84, 17.76]^b^
2024	1.67 [0.90, 3.18]	12.15 [6.80, 22.40]^c^	1.57 [0.95, 2.60]	1.90 [1.14, 3.20]^a^	3.67 [1.42, 11.37]^a^	2.51 [0.83, 9.25]
***Post-hoc:*** **Reference group set to the respective preceding academic year**						
2016–2017	1.03 [0.53, 2.03]	0.74 [0.40, 1.36]	1.45 [0.88, 2.41]	0.96 [0.58, 1.59]	1.25 [0.39, 4.34]	1.82 [0.56, 6.98]
2017–2018	0.97 [0.51, 1.86]	1.80 [1.02, 3.22]^a^	1.14 [0.71, 1.85]	0.98 [0.61, 1.59]	1.90 [0.75, 5.21]	0.60 [0.18, 1.84]
2018–2019	1.05 [0.55, 1.99]	2.05 [1.25, 3.40]^b^	2.01 [1.23, 3.31]^b^	4.08 [2.37, 7.22]^c^	1.08 [0.48, 2.41]	1.20 [0.35, 4.26]
2019–2020	1.27 [0.69, 2.36]	0.76 [0.47, 1.23]	1.33 [0.79, 2.27]	0.91 [0.49, 1.68]	1.16 [0.54, 2.51]	2.49 [0.96, 7.21]
2020–2021	0.93 [0.51, 1.68]	1.19 [0.74, 1.92]	1.07 [0.62, 1.85]	0.36 [0.21, 0.62]^c^	0.46 [0.18, 1.08]	0.98 [0.44, 2.15]
2021–2022	1.07 [0.60, 1.92]	3.94 [2.41, 6.55]^c^	0.36 [0.22, 0.60]^c^	1.45 [0.89, 2.36]	2.50 [1.10, 6.22]^a^	1.29 [0.62, 2.72]
2022–2023	1.34 [0.77, 2.35]	0.70 [0.41, 1.17]	0.89 [0.56, 1.42]	1.00 [0.61, 1.66]	1.10 [0.56, 2.17]	1.24 [0.63, 2.45]
2023–2024	0.93 [0.54, 1.62]	1.78 [1.03, 3.13]^a^	1.01 [0.63, 1.64]	1.03 [0.61, 1.74]	0.97 [0.49, 1.94]	0.50 [0.22, 1.06]

Odds ratios with confidence intervals are displayed for all post-hoc analyses, with relevant significance levels indicated.

^a^*p* < 0.05; ^b^*p* < 0.01; ^c^*p* < 0.005.

## Discussion

We engage first-year veterinary students in this self-assessment activity as we introduce the concept of “communication” as a professional competency. We use the learning outcomes through completing this assessment to identify emphasis points for subsequent (VM 2–4 year) academic curriculum directions. The advantages of this assessment are ease of access and completion, no financial costs, uncomplicated language (in English), efficient time utilization, and relevance to framing future educational discussions related to the medical interview and interpersonal communication.

During subsequent follow-up discussion students are taught the overall tendency for people, when feeling unsafe in a dialogue, to default to “silence.” We use students' own default preferences to help them understand others' similarities and differences in communication preferences. After reviewing this assignment students are provided specific techniques such as open-ended questioning and compassion phrasing in the third-year core communication course to intentionally establish a “safe” conversation dynamic so that owners will share pertinent information during the medical interview encounter (15–22).

It is interesting, but not unexpected, that most students were assigned a “silence” style through their answers to the posed questions. Given that the percentage of veterinary students recording “silence” as a default strategy in our cohorts is consistently greater than 90%, it seems clear that defaulting to a “silence” strategy is generally common and preserved response.

Within the “silence” general category, there were significant increases in all three subcategories compared to the 2016 cohort as a reference group. Of these subcategory silence groups, the “masking” group had the most significant increase over time. “Masking” is defined in the CC model as “understating or selectively showing our true opinions.” Sarcasm, sugarcoating, and couching are some of the more popular behaviors used. In essence this is communicating something that one does not believe to be true to avoid perceived negative consequences related to that belief. This strategy is a type of deflection (that is, a defense mechanism characterized by redirecting a conversation away from a challenging topic or issue to something less emotionally charged). It is a common verbal strategy of saying something while avoiding the individual's truth. The subcategories of “avoiding” and “withdrawing” were also significantly increased compared to the 2016 Cohort. These are also traditional silence strategies.

Based on these results it is reasonable to conclude that individuals are generally reluctant to willingly share their personal beliefs, feelings, perspectives, and opinions unless they feel safe in sharing this information. Students should understand this default safety strategy for themselves and as they begin to engage in medical communications with owners and producers in the future stages of their veterinary career. This tendency may also reflect other communication strategies such as conflict avoidance and have relevant in numerous veterinary communication dynamics (25).

While the silence strategy is prevalent and preserved, the percentage of students defaulting to a “violence” strategy also did not significantly change over time. In individual years, however, there was a higher percentage of students assigned in this category. Even though there was a slight trend in defaulting to a “violence” communication strategy in the last 2 years (2023–2024) as compared to the first 2 years (2016–2017) of data collection, this difference was not statistically significant.

Overall, the “violence” default is uncommon, but may appear to be more common in the based on overall public discourse and depending on which communication channel an individual accesses or is exposed. When compared to the 2016 as a reference there were increases in the “labeling” and “attacking” subcategories within the “violence” general group, however, this change did not reach statistical significance.

In analyzing our results, it is acknowledged that there are limitations to using a any similar self-reflective instrument including the “Style Under Stress” self-assessment. Limitations include individual inconsistencies in interpreting and responding to the prompts, the inability of individuals to accurately self-assess, and the lack of serious engagement with the instrument or the process (26–39). This assessment is not, nor intended to be, a validated personality or similar assessment such as the Big Five, DiSC^®^, or Myers Briggs but rather more of a training exercise, “thought-provoker,” and “conversation starter.” Additionally, the dynamics of an individual interaction may result in the adoption of a range of communication strategies not clearly articulated through the current instrument or other similar instruments and this instrument does not reflect this range of potential communication preferences. As individual strategies for engaging in crucial conversations may be influenced by innumerable variables, no single instrument can capture the diversity of potential responses that may occur in a given communication dynamic.

Additionally, and importantly, as stated in the introduction, the original research and the perspectives upon which this model developed were collected from United States multinationals relative to a United States work context and is most applicable in English-speaking environments^1^. While contextual in the United States and/or English-speaking communication environment, the basic principles within this model require cultural adaptation and attention to cultural norms in application. The cultural context should be acknowledged when applying these concepts globally and with an appreciation of cultural background, lived experience of those involved in the interaction and communication norms (13).

Given these known limitations, the broad outcomes and utility of this assessment as a learning tool and the collective results of multiple years of student responses in a US veterinary college are consistent with what is described in the CC model. As these general communication defaults apply not only to the veterinarian but, by extension, likely to owners, clients, or agents, the ramifications of these safety defaults relative to gathering accurate information in a medical interview are relevant and significant. The importance and consequences of recognition of defaulting to a “silence” strategy have been described previously in a medical context (40). This study emphasizes why this self-knowledge and developing strategies for engaging in difficult conversations is vital for both patient safety as well as the safety of the health care delivery team.

This assignment for first-year veterinary students provides not an endpoint but rather an entry point into the broader learning related to effective medical communication strategies that occur throughout the curriculum, both explicitly as well as implicitly. The results from our student cohorts and their engagement with this instrument helps to frame content in the veterinary medical communication core course delivered in the VM 3 (preclinical) year of our curriculum. We discuss the tendency for individuals, when feeling unsafe in a communication dynamic, are more likely to not share important information as they default to a “silence” strategy when unsafe. We use this concept of “style under stress” when discussing and teaching the importance of establishing rapport and most critically, “trust” in the communication dynamic between the medical professional and the client in order to counter this default to silence. The CC model also provides a unique lexicon to frame communication nuances that are difficult to articulate and, therefore, provides a common language to engage in productive dialogue.

While not the intent of this assignment, the CC model also describes strategies to move from either “style under stress” default to a more effective dialogue dynamic. These strategies include suggestions for individuals to create “safe” communication environments and equally for individuals to create safety for themselves. Some of these strategies include actions such as asking questions, active listening, and phraseology to express different perspectives. We discuss these strategies as specific skills in the medical communication course in year three of our veterinary curriculum.

In summary, “Silence” is a communication strategy used when disagreement cannot be verbalized or if there is a fear of retaliation for one's opinions, perspectives, and ideas. As medical professionals, we should all be listening to the “silence” in our colleagues, students, staff, and clients as a message to our effectiveness as communicators and health professionals. As educators, we should be mindful of these student perspectives. Finally, it is critical that we role model safe communication in our interactions to help our students develop these skillsets. We all have a responsibility in the communication dynamic to make it “safe” to have effective dialogue and ultimately provide the best health care experience possible.

## Data Availability

The original contributions presented in the study are included in the article/supplementary material, further inquiries can be directed to the corresponding author.

## Footnotes

1
https://cruciallearning.com/
